# High burden of cryptococcal antigenemia and meningitis among patients presenting at an emergency department in Maputo, Mozambique

**DOI:** 10.1371/journal.pone.0250195

**Published:** 2021-04-26

**Authors:** Robert Deiss, Carolina V. Loreti, Ana G. Gutierrez, Eudoxia Filipe, Milton Tatia, Sheila Issufo, Iza Ciglenecki, Anne Loarec, Henriques Vivaldo, Carmen Barra, Carolina Siufi, Lucas Molfino, Natalia Tamayo Antabak

**Affiliations:** 1 Médecins Sans Frontières, Maputo, Mozambique; 2 Division of Infectious Diseases, Department of Medicine, University of California, San Diego, California, United States of America; 3 HIV Programme, Ministry of Health (MoH), Maputo, Mozambique; 4 Hospital Geral José Macamo, Maputo, Mozambique; 5 Médecins Sans Frontières, Geneva, Switzerland; National Institute for Communicable Disease (NICD), South Africa, SOUTH AFRICA

## Abstract

**Background:**

Cryptococcal meningitis is a leading cause of HIV-related mortality in sub-Saharan Africa, however, screening for cryptococcal antigenemia has not been universally implemented. As a result, data concerning cryptococcal meningitis and antigenemia are sparse, and in Mozambique, the prevalence of both are unknown.

**Methods:**

We performed a retrospective analysis of routinely collected data from a point-of-care cryptococcal antigen screening program at a public hospital in Maputo, Mozambique. HIV-positive patients admitted to the emergency department underwent CD4 count testing; those with pre-defined abnormal vital signs or CD4 count ≤ 200 cells/μL received cryptococcal antigen testing and lumbar punctures if indicated. Patients with CM were admitted to the hospital and treated with liposomal amphotericin B and flucytosine; their 12-week outcomes were ascertained through review of medical records or telephone contact by program staff made in the routine course of service delivery.

**Results:**

Among 1,795 patients screened for cryptococcal antigenemia between March 2018—March 2019, 134 (7.5%) were positive. Of patients with cryptococcal antigenemia, 96 (71.6%) were diagnosed with CM, representing 5.4% of all screened patients. Treatment outcomes were available for 87 CM patients: 24 patients (27.6%) died during induction treatment and 63 (72.4%) survived until discharge; of these, 38 (60.3%) remained in care, 9 (14.3%) died, and 16 (25.3%) were lost-to follow-up at 12 weeks.

**Conclusions:**

We found a high prevalence of cryptococcal antigenemia and meningitis among patients screened at an emergency department in Maputo, Mozambique. High mortality during and after induction therapy demonstrate missed opportunities for earlier detection of cryptococcal antigenemia, even as point-of-care screening and rapid assessment in an emergency room offer potential to improve outcomes.

## Introduction

Cryptococcal meningitis (CM) is a leading cause of HIV-related mortality, causing approximately 223,000 deaths annually and also accounting for 15% of all AIDS-related deaths [1]. In Mozambique, where HIV prevalence among adults (12.6%) is eighth highest in the world, approximately 54,000 people die from AIDS-related illnesses each year [2]. Furthermore, Mozambique has the third-highest global incidence of CM, with an estimated 18,300 cases annually [1]. Data supporting these estimates, however, are sparse, and there are no published laboratory-based surveys of cryptococcal antigenemia (CrAg) in Mozambique.

A number of factors contribute to the high mortality of CM. At a treatment level, these include poor access to effective amphotericin-based treatment regimens, inadequate monitoring of drug toxicity and difficulties in the management of elevated intracranial pressure (ICP). In sub-Saharan Africa, laboratory capacity is frequently insufficient for the burden of disease and is further hampered by the lack of available rapid diagnostics. At a population level, patients often present late in the course of CM, potentially the result of difficulties in linkage and/or retention in care [3]. Likewise, inadequate follow-up care after diagnosis and treatment can itself contribute to mortality among patients with CM.

As serum cryptococcal antigenemia (CrAg) typically precedes the development of CM, it has been independently associated with decreased survival among patients with advanced HIV [4]. For this reason, the World Health Organization (WHO) recommends screening for CrAg among all individuals with a CD4 count ≤ 100 cells/μL [5]. These recommendations are supported by a number of single-country studies from sub-Saharan Africa, most of which found either cost-savings or decreased mortality when CrAg screening programs were introduced [6–13]. CrAg screening among patients with CD4 count ≤ 200 cells/μL, combined with a short intervention of community support, also substantially reduced mortality in a randomized controlled study at six sites in Zambia and Tanzania [14]. Last, a systematic review of pooled prevalence from 60 studies concluded that using a higher threshold of 200 cells/μL is warranted, as one-fifth of all cases were found among individuals with CD4 counts between 101–200 cells/μL [15]. Routine CrAg screening, however, has not been widely implemented for patients in resource-limited settings, even though available point-of-care testing can facilitate rapid diagnosis of CM and identify cryptococcal infection before the development of meningitis [16].

Beginning in March, 2018, Médecins Sans Frontières (MSF) and the Mozambican Ministry of Health (MoH) incorporated cryptococcal antigen screening into the emergency department of Jose Macamo General Hospital, a public hospital in Maputo, Mozambique. Here, we report the prevalence and associated characteristics of CrAg and CM among patients who underwent CrAg screening between March, 2018 and March, 2019, along with in-hospital and 12-week outcomes for patients diagnosed with CM.

## Methods

### Program setting and population

Point-of-care screening was incorporated into the routine clinical evaluations of patients in the emergency department (ED) at Jose Macamo General Hospital (JMGH). Patients arriving at JMCH were received in a triage area and admitted to the emergency department if they had a medical complaint requiring urgent evaluation; upon admission, they also received rapid HIV testing if their status was unknown. On weekdays between 8:00 am– 4:00 pm, nurses reviewed medical data and performed phlebotomy for HIV-positive patients who were present in the emergency department. Patients arriving overnight were generally held in the ED until the morning, and typically they were able to receive point-of-care screening prior to receiving their disposition.

As part of our initial assessment bundle, all HIV-positive patients were screened to determine CD4 count (Pima™ Analyser, Abbott, Inc.; San Diego, CA, USA); if CD4 count was less than 200 cells/uL, patients received further point-of-care screening for cryptococcal antigenemia and tuberculosis, using the Cryptococcal Antigen (CrAg) Lateral Flow Assay (IMMY; Norman, OK, USA) and Determine TB-LAM™ test (Abbott Inc.; San Diego, CA, USA), respectively. Patients also received serum CrAg and TB-LAM testing, irrespective of CD4 count, if they had any of the following abnormal vital signs: heart rate ≥ 120 beats per minute, respiratory rate > 30, oxygen saturation < 90%, systolic blood pressure < 90 mmHg or altered mental status. All laboratory testing was performed on-site, and results were available to clinicians to assist with medical management.

### Diagnosis and treatment

Patients with cryptococcal antigenemia received a lumbar puncture, and cerebrospinal fluid (CSF) was tested for the presence of CrAg using the same assay. Individuals who were serum CrAg+ and CSF CrAg- received pre-emptive antifungal therapy in accordance with WHO guidelines [5]. Patients who were diagnosed with CM received WHO-recommended regimen of liposomal Amphotericin B (3–5 mg/kg/day) and oral Flucytosine (100 mg/kg/day, divided into four doses per day) for seven days, followed by an additional seven days of either IV or orally-administered Fluconazole (1200 mg/day). For patients with pre-existing kidney disease, the alternate WHO regimen of Fluconazole (1200 mg/day administered IV) and oral Flucytosine (100 mg/kg/day) was provided during the first fourteen days of treatment [5]. Patients receiving liposomal Amphotericin B received pre-hydration including normal saline (0.9% 1000 mL), potassium chloride (26.8 mEq) and magnesium sulfate (2 grams) with each dose. Serum creatinine and potassium levels were monitored for patients receiving liposomal Amphotericin B on Days 1, 4 and 7; therapeutic lumbar punctures were performed to relieve symptoms at the discretion of treating physicians. Patients were discharged once clinically stable, with hospital stays typically lasting between 7–14 days during treatment induction.

At the time of discharge, patients completing induction treatment were referred to Centro Referencia de Alto Mae (CRAM), a specialized outpatient health center managing patients with advanced HIV infection. CRAM is located approximately four kilometers away from JMGH and is an MoH-sponsored referral center, established in 2010 in collaboration with MSF. At CRAM, referred patients continued to receive antifungal therapy (Fluconazole 800 mg/day for 10 weeks, followed by Fluconazole 200 mg/day) and ongoing monitoring and management of ICP. Antiretroviral therapy (or switch to second-line regimen) was initiated a minimum of four weeks after CM diagnosis in accordance with WHO guidelines [5].

### Data collection and analysis

We collected demographic, history, clinical and laboratory data for all patients who were screened for cryptococcal antigenemia. At the time of initial clinical and laboratory evaluation, patients were assigned a unique identifier, and this identifier was used to record clinical data, laboratory results and treatment outcomes. Data was collected prospectively as part of routine clinical care; subsequent analyses were performed retrospectively on this data. Clinical staff abstracted data from emergency department medical records (paper), and counsellors were responsible for recording outcomes, including hospital admission, discharge, death and follow-up care. As part of routine service delivery, counsellors also conducted telephone outreach for patients diagnosed with CM to ensure proper linkage to care, and these outcomes were updated using each patient’s unique identifier. Patient-level data was coded, anonymised and entered into a standardized database (Microsoft Excel 2010). All records were stored in a locked cabinet in an office outside of any clinical activity.

For this report, we analysed data collected between March 15, 2018 –March 14, 2019. Participant characteristics were summarised using frequencies, median and interquartile range [IQR], while prevalence estimates were calculated as the number of patients with CrAg and CM divided by the total number of patients who underwent CrAg screening. Chi-square and Mann-Whitney tests were used to compare several HIV-related characteristics from individuals who were serum CrAg-, serum CrAg+, serum CrAg+/CSF CrAg+ and serum CrAg+/CSF CrAg-. All analyses were performed using SPSS 26.0 (IBM).

As an *a posteriori*, secondary analysis of routinely collected clinical data from a de-identified dataset of an existing program, this study was deemed exempt from MSF ERB and Mozambican Ethics Review Board (ERB) review. All data were collected for purposes of patient’s follow-up and program monitoring. Confidentiality was respected and only de-identified data were used for the analysis. As such, the study did not require review by Mozambican ERB and it fulfilled the exemption criteria set by the MSF Ethics Review Board.

## Results

During the reporting period, a total of 2,210 HIV-positive patients in the emergency department received clinical and laboratory assessments, of whom 1,795 (81.2%) met criteria for CrAg screening. The prevalence of CrAg was 7.5% (n = 134); among patients who received an LP (n = 126), 96 (76.2%) were diagnosed with CM, yielding a prevalence of 5.3% among all screened patients. Data is presented for 1,600 patients, for whom clinical data (beyond laboratory testing) was recorded.

Selected characteristics and comparisons between serum CrAg+ and serum CrAg- individuals are highlighted in Table 1. The median age of both groups was 38 [Interquartile Range (IQR) 32–46]; neither men nor women were statistically overrepresented. Most patients were ART treatment-experienced (73.4%); approximately half reported current ART use. Median CD4 count (cells/μL) was significantly lower (p<0.001) among serum CrAg+ (34 [IQR (17, 82)] than serum CrAg- patients (84 [IQR (33, 198)]; the two groups did not significantly differ in terms of exposure to ART (73.5% vs 72.4%, p = 0.849) or years receiving therapy (2.8 [IQR 0.5, 5.8] vs 2.4 IQR [0.2, 5.5]; p = 0.571). We did not observe significant differences between CSF CrAg+ and CrAg- patients, though the numbers available for comparison were small. ART history was not available for 86 (5.4%) of patients, and CD4 count was not recorded for 27 (1.7%) of patients. Overall, 53 CM patients received TB-LAM screening, and 5 positive cases were identified.

**Table 1:** Baseline characteristics and demographics of patients with cryptococcal antigenemia and meningitis.

	Screened patients	Serum CrAg-	Serum CrAg+	P-value^1^	CSF CrAg+	CSF CrAg-	P-value^1^
	N = 1,600	N = 1,466	N = 134		N = 96^2^	N = 30^2^	
**Median years of age [IQR]**	38 [32, 46]	38 [32, 46]	38 [34, 44]	0.835	38 [33, 44]	38 [30, 42]	0.496
--Missing	69 (4.3%)	62 (4.2%	7 (5.2%)		4 (4.2%)	2 (2.1%)	
**Sex**							
--Male	732 (45.8%)	663 (45.2)	69 (51.5%)	0.163	50 (54.3%)	12 (40%)	0.161
--Female	868 (54.2%)	803 (54.8%)	65 (48.5%)		46 (45.7%)	18 (60%)	
--Missing	0 (0.0%)	0 (0.0%)	0 (0.0%)		0 (0.0%)	0 (0.0%)	
**Median CD4 count (cells/μL)**	79 [31, 193]	84 [33, 198]	34 [17, 82]	<0.001	32 [16, 70]	45 [16, 138]	0.220
--Missing	27 (1.7%)	22 (1.5%)	5 (3.7%)		4 (4.2%)	2 (2.1%)	
**Ever received ART**							
--Yes	1,174 (73.4%)	1,077 (73.5)	97 (72.4%)	0.849	65 (67.7%)	24 (80%)	0.268
--No	340 (21.3%)	313 (21.4%)	27 (20.1%)		26 (27.1%)	4 (13.3%)	
--Missing	86 (5.4%)	76 (5.2%)	10 (7.5%)		5 (5.2%)	2 (6.7%)	
**Currently receiving ART**							
--Yes	859 (53.7%)	786 (53.6%)	73 (54.5%)	0.713	50 (52.1%)	18 (60.0%)	0.294
--No	655 (40.9%)	604 (41.2%)	51 (38.1%)		41 (42.7%)	10 (33.3%)	
--Missing	86 (5.4%)	76 (5.2%)	10 (7.5%)		5 (5.2%)	2 (6.7%)	
**Median years on ART**	2.8 [0.5, 5.8]	2.8 [0.5, 5.8]	1.4 [0.2, 5.5]	0.571	0.9 [0.1, 5.0]	2.4 (0.4, 5.9)	0.275
--Missing	873 (54.6%)	807 (55.0%)	66 (49.3%)		46 (47.9%)	12 (40.0%)	
**TB-LAM positive**							
--Yes	170 (10.6%)	163 (11.1%)	7 (5.2%)	0.023	5 (5.2%)	2 (6.7%)	0.661
--No	698 (43.6%)	633 (43.2%)	65 (48.5%)		48 (50.0%)	13 (43.3%)	
--Missing	732 (45.8%)	670 (45.7%)	62 (46.3%)		43 (44.8%)	15 (50.0%)	

^1^P-Values reflect comparison between values in the preceding two columns; values ≤ 0.050 were considered statistically significant.

^2^Eight patients who were CrAg+ did not receive a lumbar puncture.

Abbreviations: CrAg (cryptococcal antigen); CSF (cerebrospinal fluid); ART (antiretroviral therapy).

By CD4 count, 11.5% and 8.5% of patients with CD4 ≤ 100 cells/μL had CrAg and CM, respectively; among patients with CD4 count between 101–200 cells/μL, the prevalence of CrAg and CM were 4.5% and 2.6% (Table 2). The most common symptoms/signs among patients with CM were headache (67%), altered mental status (62%), weakness (45%), vomiting (35%) and fever (27%).

**Table 2:** Prevalence of cryptococcal antigenemia and cryptococcal meningitis stratified by CD4 count^1^.

	CD4 count	CD4 count	CD4 count
	1–100 cells/μL	101–200 cells/μL	≥ 201 cells/μL
	N = 897 (%)	N = 309 (%)	N = 367 (%)
**Cryptococcal antigenemia**	103 (11.5)	14 (4.5)	11 (3.0)
**Cryptococcal meningitis**	76 (8.5)	8 (2.6)	7 (1.9)

^1^Total N (1,573) does not include 27 patients for whom CD4 values were not available, including six patients with cryptococcal antigenemia and five patients with cryptococcal meningitis.

**Fig 1:**
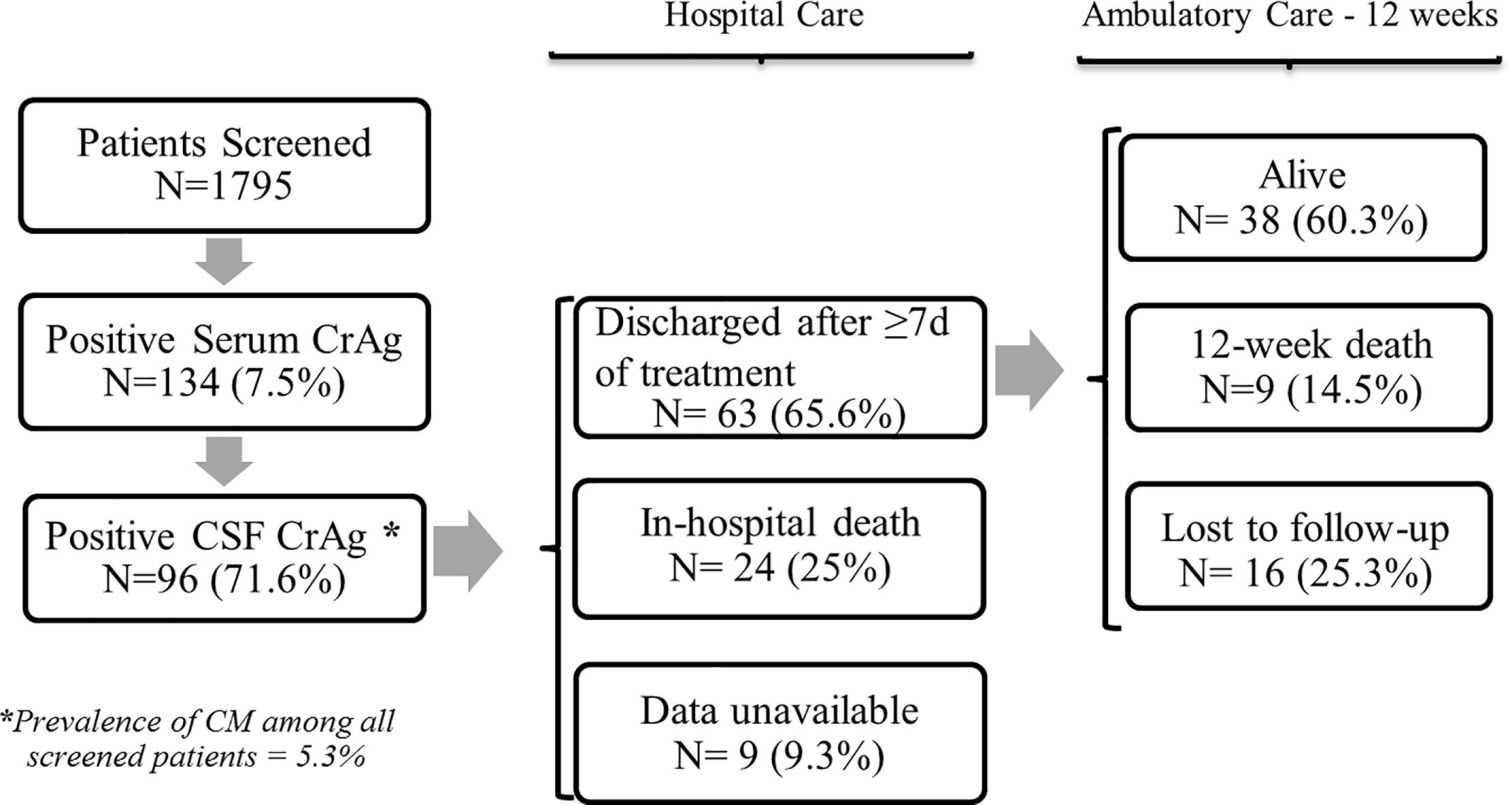
Flow diagram for patients screened and diagnosed with cryptococcal meningitis, in-hospital and 12-week outcomes.

Complete outcome data was available for 87 of 96 patients with cryptococcal meningitis (Fig 1). Among these patients, 24 (27.6%) died during their initial hospitalization, and of the remaining 63 patients, 12-week outcomes were as follows: 38 (60.3%) remained in care, 9 (14.5%) died following hospital discharge, and 16 (25.3%) were lost to follow-up. In total, 33 patients were known to have died within 12 weeks following diagnosis, constituting overall mortality of at least 37.9%.

## Discussion

In the first description of its kind, we found a high prevalence of CrAg and CM among patients presenting for emergency care in Maputo, Mozambique. In-hospital and 12-week mortality were high among patients with CM. The majority of patients diagnosed with CM was already receiving ART, indicating missed opportunities for screening at the primary health level. Overall, incorporating CrAg screening into routine emergency care for patients with advanced HIV provided a feasible means for rapid diagnosis of CrAg and CM, and point-of-care testing at this hospital continues through the present.

While data on the incidence and prevalence of cryptococcal disease in sub-Saharan Africa are sparse, [17] our estimates are consistent with one of the few systematic reviews of CM in Africa, where the average prevalence across 17 countries was 3.4% [18]. In a systematic review of the prevalence of CA, Ford et al report a combined prevalence of 6.5% and 2.0% of cryptococcal antigenemia among individuals with CD4 ≤ 200 cells/μL and between 101–200 cells/μL, respectively [15]. Our own estimates of CrAg are markedly higher, providing further support for the WHO’s conditional recommendation for screening at the higher CD4 count level, particularly among patients who are acutely ill.

A number of studies have further demonstrated the cost-effectiveness of CrAg screening, especially at lower CD4 counts [8, 10, 19, 20]. The benefit of broad CrAg screening is largely in preventing cases of CM, yet even when adding lumbar puncture and treatment of individuals newly diagnosed with CM, CrAg screening is still cost-effective [8]. Moreover, these costs are not prohibitive: Rajasingham et al developed a model, based on the incidence of CM and found that the cost of screening individuals with CD4 ≤ 100 cells/μL in most high-burden countries would be less than $1 million, or less than 0.3% of HIV budgets [1]. Even still, CrAg screening has not been a priority for international donor organizations, nor has it been widely implemented in sub-Saharan Africa, [21] as public hospitals rely most commonly on culture and India ink for diagnosis of CM.

One factor hindering CrAg screening may concern limited access to therapy once CrAg or CM are diagnosed. However, oral fluconazole is becoming increasingly available worldwide, and new treatment strategies have demonstrated promise and relevance to African contexts. The cornerstone of CM treatment remains amphotericin B, and in the recent ACTA (Antifungal Combinations for Treatment of Cryptococcal Meningitis in Africa) trial, mortality was equivalent between one-week and two-week regimens of Amphotericin B [22]. A recent Phase II clinical trial has further demonstrated the effectiveness of single, high-dose Liposomal Amphotericin B [23]. Shorter treatment courses are clearly advantageous in resource-limited settings, where in-hospital care may be limited and frequent laboratory monitoring for drug toxicity is logistically difficult. With single-dose regimens, it is conceivable that management of CM can be provided in select ambulatory or quasi-ambulatory settings, offering further rationale for CrAg and CM screening.

Even with optimal screening and adherence to treatment protocols, CM can nevertheless be severe and often fatal. Inpatient mortality (28.7%) in our sample was high and was comparable to other recent studies in sub-Saharan Africa, including the ACTA trial (2-week mortality of 21.9%) and a recent analysis of 102 cases from five South African hospitals where CrAg screening was routinely conducted [22, 24]. In this study, inpatient mortality was 30%, and the authors note that the overwhelming majority of patients were symptomatic at the time of arrival [24]. In our sample, nearly two-thirds of patients with CM presented with altered mental status, indicating significantly delayed care-seeking behaviour. In addition, 9% of patients who received TB-LAM screening were also found to have tuberculosis, which itself confers high mortality. Fang et al reported 12% mortality among PLWH who had TB/cryptococcal co-infection (disease loci not identified), [25] while a recent case series from Uganda reported 60% mortality among five patients with CM and TB meningitis [26]. Given the tendency of patients to delay care until their illness is advanced, increased CrAg screening at primary health centers is warranted to either prevent CM or diagnose it earlier.

Prevention of AIDS-related deaths also relies on early and proper identification of individuals with advanced HIV, whether it is the result of late presentation or treatment failure. Owing to earlier ART initiation and increased HIV testing, the number of late presentations in Africa has been decreasing; [27–29] in Mozambique, the proportion of patients with advanced HIV at the time of ART initiation decreased from 74 to 37% between 2004–2014 [30]. In contrast to these dramatic improvements, advanced HIV associated with treatment failure remains a significant problem [31]. Most patients in our sample reported experience with ART, and among patients with CM, one-fourth reported receiving ART for over five years. While some patients on ART were likely experiencing an unmasking illness relating to immune reconstitution, high prevalence of treatment experience among patients with CM is widely reported [32]. Clearly, earlier recognition of virologic or immunologic failure would prevent many AIDS-associated illnesses before patients arrive for hospital care, just as timely identification and rapid in-hospital assessment can improve overall outcomes.

Upon recognition of advanced HIV and provision of immediate care, an equally important element in the HIV care cascade involves linkage to and retention in care, which is challenging in many low-resource settings. Most studies on linkage to care are conducted in the context of treatment initiation following an initial diagnosis; barriers including distance, cost of travel, and weak ties between testing programs and treatment centres are commonly cited as difficulties for securing proper follow-up after HIV diagnosis [33]. Of the few studies examining linkage following a diagnosis of CM, failure to receive long-term fluconazole after discharge is the most common factor leading to either death or recurrent infection [24, 34]. Counselling patients on the need for long-term antifungal treatment is also critical, as inadequate explanation by healthcare workers was also cited as a reason for recurrent infection in the study by Quan et al. [24].

Here, an important element of the MSF/MOH collaboration is the presence of a specialized referral center (Centro Referencia de Alto Mae, abbreviated as CRAM), where counsellors and physicians are specifically trained in the care of patients with advanced HIV and recently diagnosed CM. At CRAM, patients recently discharged from inpatient facilities continue receiving fluconazole, along with ongoing management of elevated intracranial pressure and/or treatment re-induction where necessary. Counselors at the Jose Macamo General Hospital and CRAM maintain regular contact in order to ensure patient arrival and follow-up. In our review, even with post-discharge care, 12-week mortality following hospital discharge (14.5%) remained high, as did the number of patients lost to follow-up (17.2%). These data highlight the significant mortality risk of CM in spite of optimal management, along with the importance of a closely monitored continuum of care from hospitalization to long-term care.

Several limitations in our project should be noted. HIV testing and point-of-care screening were not performed on all eligible patients, for example those who were attended and discharged on evenings or weekend days. Thus, our prevalence estimates reflect a more ill population than would be the case if all HIV-positive patients were tested. Similarly, not all patients in the sample received TB-LAM screening for disseminated tuberculosis, and therefore, co-infection may be under-estimated among severely ill patients. Self-reported ART histories were subject to recall bias, and these data should be interpreted cautiously. Many patients were lost to follow-up, and thus true 12-week mortality is likely under-estimated. Last, many clinical records were incomplete, reflecting the difficulties of data collection in real-world settings.

In conclusion, we found a high prevalence of cryptococcal meningitis among patients with advanced HIV who were screened in an emergency department of a public hospital in Maputo, Mozambique. Moreover, our implementation strategy was feasible in the context of routine clinical activities of an emergency department. CrAg screening was useful in identifying patients with CrAg and CM and provided rapid initiation of treatment, though mortality remained high among patients with CM. Further scale-up of CrAg screening, and expansion to additional hospitals along with primary health facilities, is urgently needed to further improve the care of patients with CM and advanced HIV.

## References

[1] Rajasingham R, Smith RM, Park BJ, Jarvis JN, Govender NP, Chiller TM, et al. Global burden of disease of HIV-associated cryptococcal meningitis: an updated analysis. Lancet Infect Dis. 2017;17(8):873–81. 10.1016/S1473-3099(17)30243-8 28483415 PMC5818156

[2] UNAIDS. Country Fact Sheet 2019 [Available from: https://www.unaids.org/en/regionscountries/countries/mozambique.

[3] Zolopa AR, Andersen J, Komarow L, Sanne I, Sanchez A, Hogg E, et al. Early antiretroviral therapy reduces AIDS progression/death in individuals with acute opportunistic infections: a multicenter randomized strategy trial. PloS one. 2009;4(5):e5575. 10.1371/journal.pone.0005575 19440326 PMC2680972

[4] McKenney J, Bauman S, Neary B, Detels R, French A, Margolick J, et al. Prevalence, correlates, and outcomes of cryptococcal antigen positivity among patients with AIDS, United States, 1986–2012. Clin Infect Dis. 2014;60(6):959–65. 10.1093/cid/ciu937 25422390 PMC4357818

[5] World Health Organization. World Health Organization. Guidelines for the diagnosis, prevention, and management of cryptococcal disease in HIV-infected adults, adolescents and children, March 2018: supplement to the 2016 consolidated guidelines of the use of antiretroviral drugs for treating and preventing HIV infection. 30285342

[6] Alemu AS, Kempker RR, Tenna A, Smitson C, Berhe N, Fekade D, et al. High prevalence of Cryptococcal antigenemia among HIV-infected patients receiving antiretroviral therapy in Ethiopia. PLoS One. 2013;8(3):e58377. 10.1371/journal.pone.0058377 23469276 PMC3587601

[7] Ezeanolue EE, Nwizu C, Greene GS, Amusu O, Chukwuka C, Ndembi N, et al. Geographical Variation in Prevalence of Cryptococcal Antigenemia among HIV-infected Treatment-Naïve Patients in Nigeria: A multicenter cross-sectional study. JAIDS. 2016;73(1):117. 10.1097/QAI.0000000000001048 27144527 PMC4981538

[8] Jarvis JN, Harrison TS, Lawn SD, Meintjes G, Wood R, Cleary S. Cost effectiveness of cryptococcal antigen screening as a strategy to prevent HIV-associated cryptococcal meningitis in South Africa. PloS one. 2013;8(7):e69288. 10.1371/journal.pone.0069288 23894442 PMC3716603

[9] Letang E, Müller MC, Ntamatungiro AJ, Kimera N, Faini D, Furrer H, et al., editors. Cryptococcal antigenemia in immunocompromised human immunodeficiency virus patients in rural Tanzania: a preventable cause of early mortality. Open Forum Infect Dis; 2015: Oxford University Press. 10.1093/ofid/ofv046 PMC4511744 26213690

[10] Meya DB, Manabe YC, Castelnuovo B, Cook BA, Elbireer AM, Kambugu A, et al. Cost-effectiveness of serum cryptococcal antigen screening to prevent deaths among HIV-infected persons with a CD4+ cell count≤ 100 cells/μ L who start HIV therapy in resource-limited settings. Clin Infect Dis. 2010;51(4):448–55. 10.1086/655143 20597693 PMC2946373

[11] Ogouyèmi-Hounto A, Zannou D, Ayihounton G, Ahouada C, Azon-Kouanou A, Acakpo J, et al. Prevalence and factors associated with cryptococcal antigenemia in HIV-infected patients in Cotonou/Benin. J Mycol Med. 2016;26(4):391–7. 10.1016/j.mycmed.2016.08.007 27641486

[12] Oladele RO, Akanmu AS, Nwosu AO, Ogunsola FT, Richardson MD, Denning DW, editors. Cryptococcal antigenemia in Nigerian patients with advanced human immunodeficiency virus: influence of antiretroviral therapy adherence. Open Forum Infect Dis; 2016: Oxford University Press. 10.1093/ofid/ofw055 PMC4866571 27186581

[13] Vidal JE, Toniolo C, Paulino A, Colombo A, dos Anjos Martins M, da Silva Meira C, et al. Asymptomatic cryptococcal antigen prevalence detected by lateral flow assay in hospitalised HIV‐infected patients in São Paulo, Brazil. Tropical Medicine & International Health. 2016;21(12):1539–44. 10.1111/tmi.12790 27699970

[14] Mfinanga S, Chanda D, Kivuyo SL, Guinness L, Bottomley C, Simms V, et al. Cryptococcal meningitis screening and community-based early adherence support in people with advanced HIV infection starting antiretroviral therapy in Tanzania and Zambia: an open-label, randomised controlled trial. The Lancet. 2015;385(9983):2173–82. 10.1016/S0140-6736(15)60164-7 25765698

[15] Ford N, Shubber Z, Jarvis JN, Chiller T, Greene G, Migone C, et al. CD4 cell count threshold for cryptococcal antigen screening of HIV-infected individuals: a systematic review and meta-analysis. Clin Infect Dis. 2018;66(suppl_2):S152–S9. 10.1093/cid/cix1143 29514236 PMC5850628

[16] Klausner JD, Vijayan T, Chiller T. Sensitivity and specificity of a new cryptococcal antigen lateral flow assay in serum and cerebrospinal fluid. MLO: medical laboratory observer. 2013;45(3):16. 23822028 PMC4119400

[17] Nyazika TK, Tatuene JK, Kenfak-Foguena A, Verweij PE, Meis JF, Robertson VJ, et al. Epidemiology and aetiologies of cryptococcal meningitis in Africa, 1950–2017: protocol for a systematic review. BMJ Open. 2018;8(7):e020654. 10.1136/bmjopen-2017-020654 30061436 PMC6067404

[18] Assogba K, Belo M, Wateba MI, Gnonlonfoun DD, Ossou-Nguiet PM, Tsanga BB, et al. Neuromeningeal cryptococcosis in sub-Saharan Africa: Killer disease with sparse data. Journal of neurosciences in rural practice. 2015;6(2):221. 10.4103/0976-3147.153231 25883484 PMC4387815

[19] Kimaro GD, Mfinanga S, Simms V, Kivuyo S, Bottomley C, Hawkins N, et al. The costs of providing antiretroviral therapy services to HIV-infected individuals presenting with advanced HIV disease at public health centres in Dar es Salaam, Tanzania: Findings from a randomised trial evaluating different health care strategies. PloS one. 2017;12(2):e0171917. 10.1371/journal.pone.0171917 28234969 PMC5325220

[20] Smith RM, Nguyen TA, Ha HTT, Thang PH, Thuy C, Lien TX, et al. Prevalence of cryptococcal antigenemia and cost-effectiveness of a cryptococcal antigen screening program–Vietnam. PloS one. 2013;8(4):e62213. 10.1371/journal.pone.0062213 23626792 PMC3633872

[21] Medecins Sans Frontieres. No Time to Lose: Detect, Treat and Prevent AIDS. 2019. Available at: https://msfaccess.org/no-time-lose-detect-treat-and-prevent-aids

[22] Molloy SF, Kanyama C, Heyderman RS, Loyse A, Kouanfack C, Chanda D, et al. Antifungal combinations for treatment of cryptococcal meningitis in Africa. N Engl J Med. 2018;378(11):1004–17. 10.1056/NEJMoa1710922 29539274

[23] Jarvis JN, Leeme TB, Molefi M, Chofle AA, Bidwell G, Tsholo K, et al. Short Course High-dose Liposomal Amphotericin B for HIV-associated Cryptococcal Meningitis: A phase-II Randomized Controlled Trial. Clin Infect Dis. 2019;68(3):393–401. 10.1093/cid/ciy515 29945252 PMC6336908

[24] Quan V, Toro-Silva S, Sriruttan C, Chetty V, Chihota V, Candfield S, et al. Pathways to care and outcomes among hospitalised HIV-seropositive persons with cryptococcal meningitis in South Africa. PloS one. 2019;14(12):e0225742. 10.1371/journal.pone.0225742 31830060 PMC6907845

[25] Fang W, Zhang L, Liu J, Denning DW, Hagen F, Jiang W, et al. Tuberculosis/cryptococcosis co-infection in China between 1965 and 2016. Emerg Microbes Infec. 2017;6(1):1–7. 10.1038/emi.2017.61 28831193 PMC5583669

[26] Ellis J, Cresswell FV, Rhein J, Ssebambulidde K, Boulware DR, editors. Cryptococcal meningitis and tuberculous meningitis co-infection in HIV-infected Ugandan adults. Open Forum Infect Dis; 2018: Oxford University Press US. 10.1093/ofid/ofy193 PMC6114196 30182032

[27] Gesesew HA, Ward P, Woldemichael K, Mwanri L. Late presentation for HIV care in Southwest Ethiopia in 2003–2015: prevalence, trend, outcomes and risk factors. BMC Infect Dis. 2018;18(1):59. 10.1186/s12879-018-2971-6 29378523 PMC5789710

[28] Kwobah CM, Braitstein P, Koech JK, Simiyu G, Mwangi AW, Wools-Kaloustian K, et al. Factors associated with late engagement to HIV care in Western Kenya: a cross-sectional study. Journal of the International Association of Providers of AIDS Care (JIAPAC). 2016;15(6):505–11. 10.1177/2325957414567682 25589304

[29] Mutimura E, Addison D, Anastos K, Hoover D, Dusingize JC, Karenzie B, et al. Trends in and correlates of CD4+ cell count at antiretroviral therapy initiation after changes in national ART guidelines in Rwanda. AIDS. 2015;29(1):67. 10.1097/QAD.0000000000000520 25562492 PMC4487360

[30] Auld AF, Shiraishi RW, Oboho I, Ross C, Bateganya M, Pelletier V, et al. Trends in prevalence of advanced HIV disease at antiretroviral therapy enrollment—10 countries, 2004–2015. MMWR. 2017;66(21):558. 10.15585/mmwr.mm6621a3 28570507 PMC5657820

[31] Ousley J, Niyibizi AA, Wanjala S, Vandenbulcke A, Kirubi B, Omwoyo W, et al. High Proportions of Patients With Advanced HIV Are Antiretroviral Therapy Experienced: Hospitalization Outcomes From 2 Sub-Saharan African Sites. Clin Infect Dis. 2018;66(suppl_2):S126–s31. 10.1093/cid/ciy103 29514239 PMC5850537

[32] Loyse A, Burry J, Cohn J, Ford N, Chiller T, Ribeiro I, et al. Leave no one behind: response to new evidence and guidelines for the management of cryptococcal meningitis in low-income and middle-income countries. Lancet Infect Dis. 2019;19(4):e143–e7. 10.1016/S1473-3099(18)30493-6 30344084

[33] Jani IV, Sitoe NE, Alfai ER, Chongo PL, Quevedo JI, Rocha BM, et al. Effect of point-of-care CD4 cell count tests on retention of patients and rates of antiretroviral therapy initiation in primary health clinics: an observational cohort study. Lancet. 2011;378(9802):1572–9. 10.1016/S0140-6736(11)61052-0 21951656

[34] Lessells RJ, Mutevedzi PC, Heller T, Newell M-L. Poor long-term outcomes for cryptococcal meningitis in rural South Africa. S Afr Med J. 2011;101(4):251–2. 10.7196/samj.4378 21786729

