# Correction: High burden of cryptococcal antigenemia and meningitis among patients presenting at an emergency department in Maputo, Mozambique

**DOI:** 10.1371/journal.pone.0298877

**Published:** 2024-02-08

**Authors:** Robert Deiss, Carolina V. Loreti, Ana G. Gutierrez, Eudoxia Filipe, Milton Tatia, Sheila Issufo, Iza Ciglenecki, Anne Loarec, Henriques Vivaldo, Carmen Barra, Carolina Siufi, Lucas Molfino, Natalia Tamayo Antabak

There are errors in the Funding and Competing Interests statements. The correct statements are:

**Funding:** This work was conducted with resources and funding provided by Médecins Sans Frontières (MSF) in concert with the Mozambican Ministry of Health (MoH). MSF provided support in the form of salaries for RD, CVL, AGG, IC, AL, HV, CB, CS, LM, and NTA. The specific roles of these authors are articulated in the ‘author contributions’ section. MSF programmatic funding also supported laboratory testing and database management, but the funders otherwise had no role in study design, data collection and analysis, decision to publish, or preparation of the manuscript.

**Competing Interests:** The authors have read the journal’s policy and have the following competing interests to declare: Médecins Sans Frontières (MSF) provided support in the form of salaries for RD, CVL, AGG, IC, AL, HV, CB, CS, LM, and NTA. This does not alter our adherence to *PLOS ONE* policies on sharing data and materials. There are no patents, products in development or marketed products associated with this research to declare.
